# Green and Efficient Determination of Fluoroquinolone Residues in Edible Green Fruits and Leafy Vegetables by Ultrasound-Assisted Extraction Followed by HPLC-MS/MS

**DOI:** 10.3390/molecules27196595

**Published:** 2022-10-05

**Authors:** Francesca Merlo, Dario Centenaro, Federica Maraschi, Antonella Profumo, Andrea Speltini

**Affiliations:** 1 Department of Chemistry, University of Pavia, 27100 Pavia, Italy; 2 Department of Drug Sciences, University of Pavia, 27100 Pavia, Italy

**Keywords:** drugs, emerging pollutants, green sample treatment, food matrices, food safety, cleanup, multiple reaction monitoring

## Abstract

In this work, a simple, quick and efficient analytical method for determination of human and veterinary fluoroquinolone antimicrobial residues in lettuce, cucumber and spinach is developed. The procedure entails a 6 min ultrasound-assisted extraction (UAE, 3 × 2 min) in an alkaline (2% *v/v* NH_3_) aqueous solution containing Mg^2+^ ions (3 × 6 mL), with no need for organic solvents. The extract is submitted to cleanup on the HLB™ cartridge and the fluoroquinolones are separated and quantified by HPLC-MS/MS in a 10 min chromatographic run, using a small amount of acetonitrile in the mobile phase. The method, entirely developed in real matrices, is validated according to the updated analytical guidelines and provided suitable recoveries in the range of 67–116% and precision (RSD ≤ 20%, *n* = 3) at different concentrations (15, 70 and 150 ng g^−1^), with method quantification limits of 2–10 ng g^−1^. Fluoroquinolones were detected and quantified at concentrations from few to hundreds of nanograms per gram in vegetables from supermarkets, demonstrating the applicability of the method for monitoring residues of these pharmaceuticals.

## 1. Introduction

Antimicrobials (AMs) are natural, semi-synthetic or artificial compounds, which are widely used for the treatment of infections and for disease prevention in humans and animals, and, in some countries, to promote animal growth. However, AMs are not completely metabolized and a fraction between 17% and 90% is excreted unchanged in urine and feces, leading to notably widespread contamination of water and soil resources. AMs may enter agricultural fields mainly via the use of recycled water for irrigation due to the incomplete removal of these pharmaceuticals in wastewater treatment processes, and land application of sewage sludge, biosolids and manure as soil amendments or fertilizers instead of chemical fertilizers, which are restricted for organic vegetables [1,2,3,4,5,6,7,8,9,10,11,12].

Obviously, in the context of the circular economy the agricultural applications of the reclaimed wastewaters and manure is of growing interest and importance. On the other hand, there is a considerable possibility of the introduction—via plant uptake—of AMs into crops, with the non-negligible consequences of the spread of antimicrobial resistance and/or toxic potential effects to consumers because of prolonged low-level exposure to AMs [1,2,4,6,9,10,11]. Fluoroquinolones (FQs) are one of the most employed class of AMs, adopted both for human and veterinary medicine. The intensive use of these drugs and the great chemical stability of the heterocyclic ring make them highly persistent contaminants and have led to notably widespread diffusion and environmental contamination [13]. Despite the existence of a huge database on the contamination levels in water systems throughout the world, in-depth knowledge of the activity and fate of these drugs in edible fruits and vegetables is limited.

FQs can translocate and become distributed in the sequence leaf > stem > root [6], and recent studies showed that FQs at concentrations up to several hundred ng g^−1^ were detected in different crop plants, such as solanaceous fruits, cucumbers, radishes and leafy vegetables [2,9,12,14,15]. Therefore, the monitoring of their residues in vegetables and fruits has become essential to assess human exposure, although no maximum residue levels (MRLs) are fixed for any FQ drug in edible plants. Accurate and sensitive analytical methods are required for the monitoring of residues of veterinary drugs in fruits and vegetables to ensure the safety and quality of these products.

However, the main challenge in the analysis of FQs in edible plants is related to their low concentration in vegetable tissues, and the presence of matrix components such as pigments, carbohydrates, and fatty or waxy materials can interfere in their determination [9]. The quantitative analysis can be performed by high-performance liquid chromatography equipped with fluorimetric detector (HPLC-FD) due to the high fluorescence of these compounds [16,17] and by high-performance liquid chromatography coupled with mass spectrometry detection (HPLC-MS/MS) [3,9,10,18,19], particularly recommended for complex matrices. Before instrumental quantitation, the sample preparation plays a key role in terms of analyte extraction, enrichment, and cleanup, but in most cases, it is a time-consuming and expensive step, and may contribute to further environmental problems mainly due to the quantities of hazardous wastes and the high energy demand. In this context, sample preparation techniques that meet the “Ten Principles of Green Sample Preparation” are increasingly required. However, the few methods currently proposed for FQ determination in vegetable matrices make use of prolonged extraction steps, mainly by ultrasound-assisted extraction (UAE) [9,10,12,20] and QuEChERS [2,15,21], by means of organic solvents such as acetonitrile (ACN), methanol (MeOH), acetone, hexane and chloroform. After the extraction, a cleanup step is usually performed, mostly by on-column solid-phase extraction (SPE) [9,12,18,19], dispersive SPE (d-SPE) [2,10,15,21] and liquid-liquid extraction (LLE) [20], which also require great amounts of organic solvents or sorbent material.

The aims of this paper are the development and validation of an environmentally friendly and cost-effective analytical method for the determination of FQs at low ng g^−1^ levels from leafy and fruity vegetables with high water contents. Considering the agricultural practices that make use of biosolids as fertilizers and recycled waters of different origin, both veterinary and human FQs have been selected, namely Marbofloxacin (MAR), Levofloxacin (LEV), Norfloxacin (NOR), Ciprofloxacin (CIP), Danofloxacin (DAN), Enrofloxacin (ENR) and Orbifloxacin (ORB). With respect to the available methods, the work focused on simplicity in operation, reduced consumption of organic solvents and, hence, lower waste production. The method is entirely developed in real matrices, validated, and then applied to the analysis of edible green fruits and leafy vegetables from the supermarket.

## 2. Results and Discussion

### 2.1. Development of UAE Procedure and Cleanup

The procedure consisted of two steps: UAE from freeze-dried samples and SPE purification of the extracts to remove interfering matrix components.

A first series of experiments was carried out to evaluate the cleanup conditions on HLB™ cartridges, a hydrophilic–lipophilic balanced polymer. In detail, 0.3 g freeze-dried spinach, selected as the probe matrix, was extracted according to the following preliminary conditions: 2 × 10 min UAE cycles, using 3 mL 20% *w/v* Mg(NO_3_)_2_ + 2% *v/v* NH_3_. This alkaline solution containing Mg^2+^ ions was selected in view of the extraction capability towards FQs from solid environmental matrices [5]. The merged UAE extracts (6 mL) were spiked with 7.5 ng mL^−1^ of each FQ, diluted to 25 mL with tridistilled water, and acidified at pH 3. After sample loading on HLB™ cartridges [16] and sorbent washing with 5 mL H_2_O, 5 mL NH_3_ 0.5% (*v/v*) and 5 mL H_2_O, sequentially, the elution was performed with 2 × 2.5 mL 0.1% HCOOH-ACN (80:20, *v/v*). Quantitative analysis was performed by HPLC-MS/MS in multiple reaction monitoring (MRM) mode.

These experiments showed that analytes were quantitatively recovered (73–107%, *n =* 3), and the matrix co-extracted constituents did not interfere in the MRM chromatograms, achieving an accurate quantitation. Quantitative results were also obtained with a single elution (2.5 mL) without any cartridge washing, thus minimizing the sample treatment in agreement with Green Sample Preparation Principles [22]. The good results reached by this simplified SPE cleanup are likely due to the mild conditions of UAE, in particular the use of a 100% aqueous extracting solution containing Mg^2+^ ions that imparts selectivity to the process by forming chelates with the analytes at pH above their *p*K*_a1_* (referred to the carboxylic group) [5], while reducing extraction of the less polar matrix constituents.

Once the accurate quantification by HPLC-MS/MS after the HLB™ cleanup was verified, recovery tests on freeze-dried samples enriched with 150 ng g^−1^ dry weight (d.w.) of each drug were performed to investigate the UAE efficiency in this kind of sample using the above-cited aqueous solution. Extraction efficiency was expressed as recovery (R%), calculated by the internal standard (IS) method (see Section 3.3). Under the above conditions (two UAE cycles × 10 min × 3 mL 20% *w/v* Mg(NO_3_)_2_ + 2% *v/v* NH_3_), recoveries were in the range 47–71% (*n* = 3), highlighting partial extraction from the plant matrix. To improve the UAE extraction efficiency, the influence of various parameters was evaluated, such as the number and time of sonication cycles, and the composition of extractant solution.

**Figure 1:**
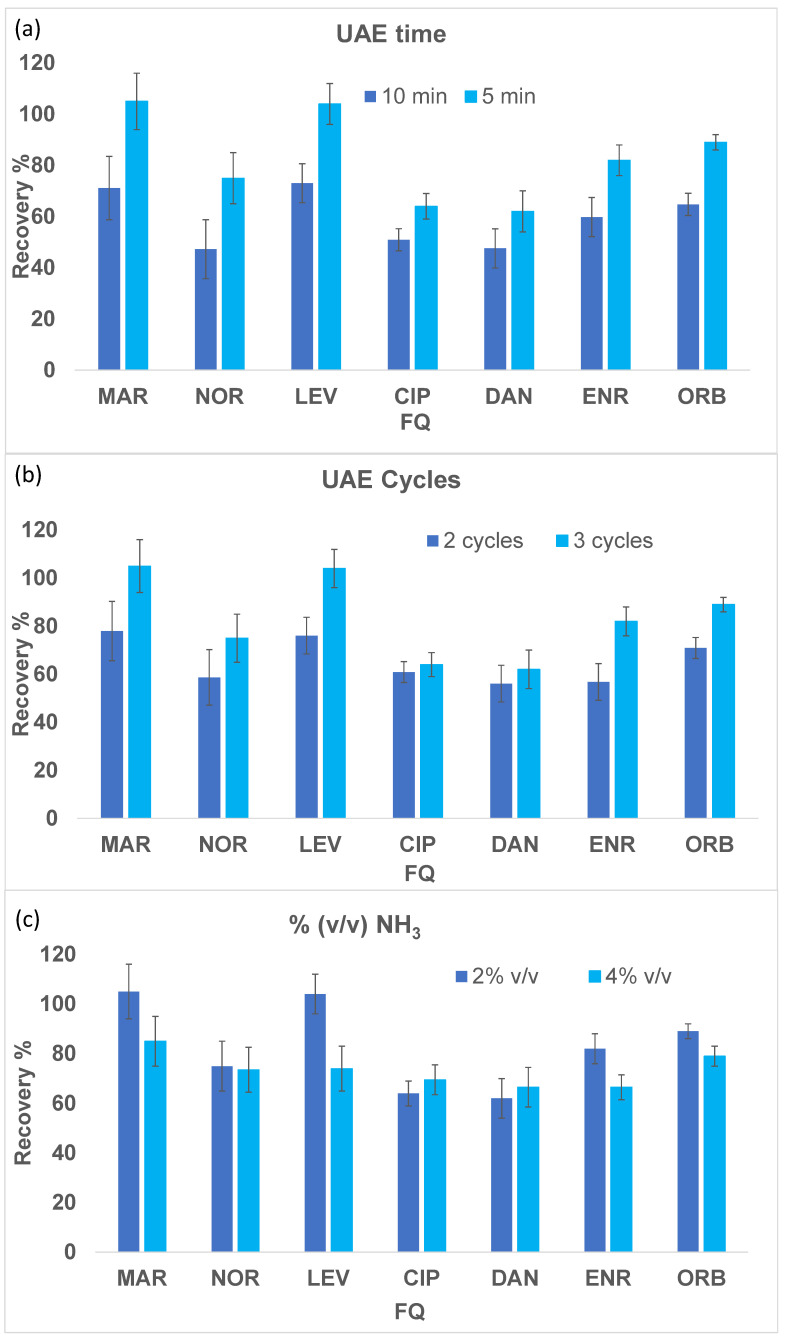
(**a**) Influence of time per UAE cycle on extraction efficiency (% R, *n* = 3) from freeze-dried spinach. (**b**) Influence of number of UAE cycle on extraction efficiency (% R, *n* = 3) from freeze-dried spinach. (**c**) Influence of ammonia amount on extraction efficiency (% R, *n* = 3) from freeze-dried spinach.

Firstly, the time per UAE cycle was studied and, as shown in Figure 1a, the reduction from 10 to 5 min did not induce statistically significant variations in recoveries, in agreement with Albero et al. [8]. On the other hand, the number of UAE cycles influenced the recovery of the analytes from the plant matrix (see Figure 1b). Increasing the cycles up to three gave the highest recovery of all the analytes, without affecting the ionization in the ESI source.

In the subsequent step, the composition of the extractant solution was studied. As anticipated above, Mg^2+^ forms chelate with the deprotonated carboxylic acid group of FQs [5], thus it is necessary to adjust the solution pH at alkaline values. The ammonia amount was checked at two levels, i.e., 2 and 4% *v/v*, with no statistically significant difference in the recoveries (Figure 1c), thus indicating that 2% *v/v* ammonia ensures a sufficiently alkaline environment during extraction.

Driven by these encouraging results, further trials were performed under selected conditions but with a sonication time of 2 min per cycle, obtaining satisfactory recoveries in the range 63–105% with relative standard deviation (RSD% < 15%, *n* = 3).

Therefore, the final procedure—three extraction cycles (2 min each) with 3 mL of Mg^2+^ aqueous solution at 2% (*v/v*) NH_3_ and cleanup on HLB™ cartridge—was extended to other edible vegetable matrices such as lettuce and cucumber, enriched at 150 ng g^−1^ of each FQ. However, the different nature of the freeze-dried samples (in terms of specific volume) made it necessary to increase the volume of the extracting solution from 3 mL to 6 mL to ensure good wettability of the matrices. Consequently, the volume of the aqueous sample submitted to cleanup on HLB™ was increased from 25 mL to 50 mL (a volume well below the cartridge breakthrough [16]), keeping the elution volume constant.

Recovery tests were performed in triplicate at three concentration levels (see Table 1), namely high-quality control (HQC, 150 ng g^−1^), medium-quality control (MQC, 70 ng g^−1^) and low-quality control (LQC, 15 ng g^−1^), which are close to the method quantification limits (MQLs, see Table 2 in Section 2.2). Recovery was calculated as the percentage of analyte extracted, quantified using the IS method, in relation to the spiking level. As reported in Table 1, for each analyte the % R values were very similar in the three samples, with satisfactory values considering the complexity of the matrices, further demonstrating that the macroscopic variability in the matrix composition among the considered vegetables/fruits does not affect the extraction efficiency.

These data account for a good UAE extraction efficiency and a quantitative recovery of the drugs throughout the overall sample preparation procedure, also at the low nanograms per gram levels, and therefore the practical applicability is assured.

To further confirm the reliability of the method, a recovery test was performed in duplicate on a freeze-dried sample fortified at the QCM level and left in the dark at room temperature for one month. The recovery values agreed with the results obtained from an equivalent sample processed 12 h after enrichment, thus demonstrating an effective adsorption of FQs to the matrix and their stability therein, excluding any photo/biodegradation.

### 2.2. Analytical Evaluation of the Method

The entire analytical procedure was within-laboratory evaluated, according to the key features described in detail in the Section 3.5.

**Figure 2:**
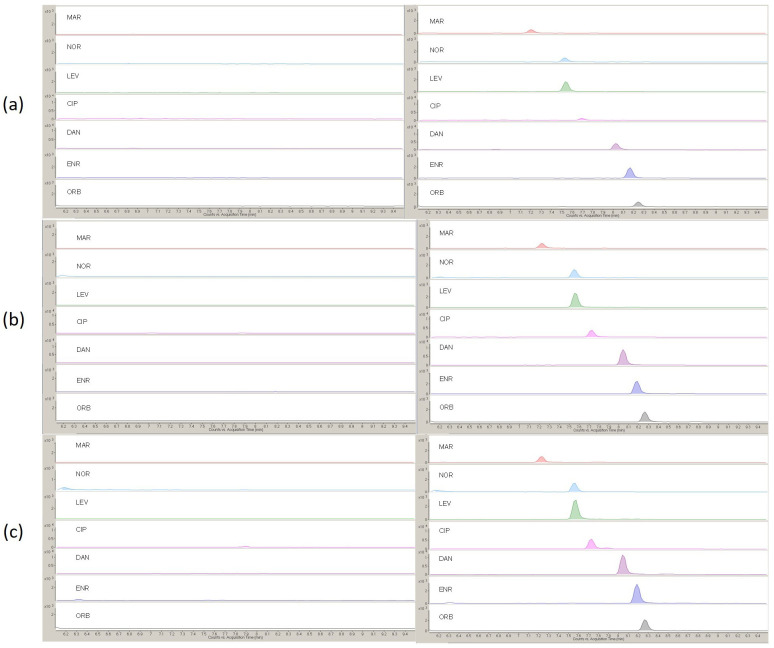
MRM chromatograms of blank sample extract and spiked sample extract (2.5 ng mL^−1^) from (**a**) spinach, (**b**) lettuce, (**c**) cucumber.

Selectivity was guaranteed by MRM detection, which affords identification/quantification of the target compounds by using the most intense transition precursor/product ions of each compound (see Table S1, Supplementary Material). Moreover, it was further corroborated based on the ability of the proposed method to detect and discriminate the target FQs from other compounds present in the sample extract; indeed, no interfering compounds were found at the retention time of the FQs when extracts from unspiked samples were compared to the same extracts spiked with 2.5 ng mL^−1^ (see Figure 2).

As stated in Tables S2–S5 (Supplementary Material), the linearity was assessed both in the pure solvent and in the purified extracts from freeze-dried food samples in the concentration range of 2–25 ng mL^−1^, with adequate coefficients of correlation (R^2^> 0.9920, *n* = 3).

Table 2 reports the method detection limits (MDLs) and method quantification limits (MQLs) obtained from each matrix-matched calibration curve for all the analytes, taking into consideration the whole sample treatment. No MRLs have been established for these antimicrobials in vegetables and fruits, however, the herein obtained limits underline the applicability of the proposed method for FQ contamination monitoring, from few to hundreds ng g^−1^.

**Figure 3:**
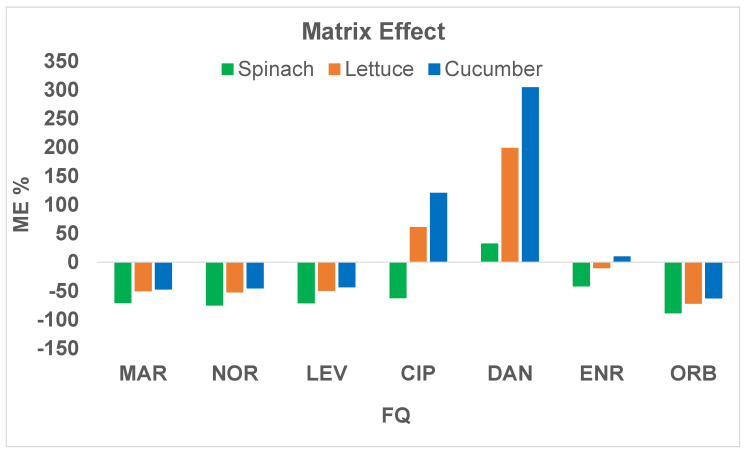
Matrix effect (ME %) for each matrix.

The presence of matrix effect (ME) was assessed by comparing the slopes of matrix-matched calibration curves with those of the calibration lines obtained in the pure solvent, achieving the results reported in Figure 3. It is possible to witness a suppression of the signal (negative ME) for almost all the analytes, except for DAN and CIP.

Based on the considerable matrix effect, Enoxacin (ENOX) was used as the IS to correct the suppression/enhancement of ionization since, belonging to the class of FQs, it is a structural analogue of the target analytes, but its use has been banned for years.

The extraction efficiency of the method was confirmed through recovery tests using freeze-dried samples spiked at HQC, MQC and LQC, achieving trueness values (see Table 1) that fall within the ranges of 80–110% (for concentrations from 1.0 to 0.1 µg g^−1^, as HQC) and 60–115% (for levels between 0.01 and 0.1 µg g^−1^, as MQC and LQC), with inter-day precision (RSD ≤ 20%, *n* = 3), in full agreement with criteria for analytical methods for food sample monitoring [23].

The entire procedure turned out to be robust since small deliberate variations (e.g., volumes for SPE conditioning, ultrasound bath temperature ± 5 °C, sample pH ± 0.2 before SPE, etc.) did not cause appreciable changes in the accuracy.

The repeatability of the injection was checked injecting in the HPLC-MS/MS a matrix standard at 15 ng mL^−1^. The % RSD value associated with the peak area by repeated injections (*n* = 10) was below 5%, both in the case of the pure solvent and in the SPE eluate from each vegetable.

No instrumental carry-over was observed in the MRM chromatogram of the pure solvent and blank extracts injected between either the consecutive sample from the recovery tests or after injection of the highest calibration standard.

### 2.3. Comparison of the Developed Procedure with Recent UAE-Based Methods in Edible Green Fruits and Leafy Vegetables 

The final procedure represents a neat improvement compared to the few literature methods, developed in recent studies, entailing UAE coupled to SPE cleanup. As clearly shown in Table 3, these methods make use of time-consuming extractions and large volumes of organic solvents, in contrast to the herein proposed UAE based on an alkaline aqueous solution of magnesium ions, with a similar or even higher sensitivity.

### 2.4. Determination of FQs in Commercial Green Fruits and Leafy Vegetables

The final procedure was used to investigate the occurrence of target FQs in commercial edible samples. In detail, spinach, lettuce and cucumber were purchased in a supermarket located in Pavia (Italy), lyophilized (see Section 3.2), and submitted to the final method (see Section 3.3 and Section 3.4 ). Generally, the antimicrobials were quantified at concentration levels of few ng g^−1^ or lower than MQLs. In detail, CIP was found at 6 and 4 ng g^−1^ in spinach and lettuce, respectively. ENR was quantified at 5 and 4 ng g^−1^ in cucumber and lettuce, respectively. Cucumber also showed the presence of LEV at 4 ng g^−1^ and of NOR at 130 ng g^−1^. A representative MRM chromatogram is shown in Figure S1 (Supplementary Material), referred to as the cucumber sample. These results are consistent with the recent literature data about contamination levels in edible plants (root and leafy vegetables), where these drugs were quantified in a wide concentration range, from few to hundreds ng g^−1^ [2,9,12,14,15]. 

Moreover, these experimental data confirm the ability of plants to absorb antimicrobials from the environment and store them in leaves and fruits [1].

## 3. Materials and Methods

### 3.1. Chemicals and Materials

All chemicals were reagent grade or higher in quality. Formic Acid (HCOOH, for ACS analysis), HPLC gradient grade MeOH, ACN, ultrapure water and anhydrous NaOH pellets (>97%) were obtained from Carlo Erba Reagents (Milan, Italy). Ammonia solution (NH_3_, 30%, *v/v*) and hexahydrate Mg(NO_3_)_2_ (97%) were supplied by Sigma Aldrich (Milan, Italy). Nylon filters (0.45 µm, 25 mm) were supplied by Sharlab S.L. (Barcelona, Spain), whereas Nylon filters (0.22 µm, 13 mm), 1 mL and 5 mL syringes were provided by VWR International Srl (Milan, Italy). Polypropylene tubes (10 and 50 mL) were provided by Tecnovetro (Varedo, Italy). Oasis^®^ HLB™ (200 mg) cartridges were purchased from Sigma Aldrich (Milan, Italy).

CIP was purchased from Alfa Aesar (Kandel, Germany), Marbocyl^®^ was provided by Vetoquinol (Bertinoro, Italy) as a solution (2%) for injection, ENR was obtained from Tokyo-Chemical-Industry, LEV, NOR and ENOX were supplied by Sigma Aldrich (Milan, Italy), and DAN and ORB were provided by Fluka (Milan, Italy).

FQ stock solutions of 300 mg L^−1^ were prepared in MeOH containing 0.1% *v/v* 1 M NaOH and stored in the dark at 4 °C for a maximum of three months. Multiple solutions were prepared in 0.1% *v/v* HCOOH-ACN (80:20, *v/v*) or MeOH containing all the analytes at different concentrations (6 mg L^−1^, 480 ng mL^−1^, 240 ng mL^−1^, 120 ng mL^−1^).

### 3.2. Edible Green Fruits and Leafy Vegetable Samples

Spinach (*Spinacia oleracea*) was selected for the development of the extraction and cleanup procedure. The final method was validated also on lettuce (*Lactuca sativa* L.) and cucumber (*Cucumis sativus* L.).

Freeze-dried spinach was provided by Good Smoothie GmbH (Böhl-Iggelheim, Germany), whereas fresh lettuce and cucumber samples were purchased in a supermarket located in Voghera (Italy). These fresh vegetables were cut into small pieces, stored in a deep-freezer container at −20 °C, and then submitted to the freeze-drying process (2 days or longer) by using a Lio 5P (5Pascal) instrument to remove residual water without loss of food quality [24].

### 3.3. UAE Procedure and Cleanup

UAE (45 kHz, 80 W USC 200–2600 ultrasonic cleaner, VWR, Milan, Italy) was performed on lyophilized samples (0. 25 g d.w., 0.5 g d.w. for spinach). For recovery tests, samples were transferred into watch glasses, spiked with each analyte to reach the final concentration (15–150 ng g^−1^ d.w. of each analyte), manually homogenized in the watch glass and stored in the dark for approximately 12 h at room temperature to allow the evaporation of the residual MeOH. The samples were transferred into 50 mL Falcon Tubes for the extraction. The latter involves 3 × 2 min UAE cycles, with 6 mL of an aqueous solution of 20% *w/v* Mg(NO_3_)_2_ + 2% *v/v* NH_3_. After sonication, suspensions were centrifuged for 5 min (4500 rpm for spinach, 8000 rpm for lettuce and cucumber). To reduce ME resulting from matrix co-extracted components, the merged UAE extracts were diluted to 50 mL with tridistilled water, acidified at pH 3 with HCl and preconcentrated on HLB™ cartridges by means of a multi-position vacuum manifold (Resprep manifold, Restek Corporation, Bellefonte, USA). The cartridge was previously conditioned with 5 mL MeOH, 5 mL distilled water and 5 mL 0.1% *v/v* HCOOH. After sample loading (~5 mL min^−1^), the sorbent bed was dried under strong vacuum for 2 min. The elution was performed with 2.5 mL 0.1% HCOOH-ACN (80:20, *v/v*) at ~0.5 mL min^−1^, and the eluate was spiked with 9 ng mL^−1^ ENOX as the IS and directly analyzed by HPLC-MS/MS (MRM mode).

### 3.4. HPLC-ESI-MS/MS

The instrumental analysis was performed with an Agilent (Cernusco sul Naviglio, Italy) HPLC apparatus 1260 Infinity coupled with an Agilent 6460C MS spectrometer ESI-MS/MS system.

An Agilent (Cernusco sul Naviglio, Italy) 120 EC-C18 Poroshell column (3 × 50 mm, 2.7 µm) equipped with a similar guard column was used as analytical column thermostated at 25 °C (±0.8 °C). The elution conditions were step-by-step refined to ensure a suitable separation of the selected FQs in the shortest time. The final gradient elution (0.5 mL min^−1^) was performed by (A) 0.05% (*v/v*) HCOOH and (B) ACN, according to the following program: 5% B for 0.5 min, linear gradient to 40% B in 8.5 min, linear gradient to 98% B in 0.5 min, hold for 2 min (washing step); the initial conditions (5% B) were returned by 7.5 min equilibration time. The sample injection volume was 10 μL, robotically injected by the HPLC autosampler. Typical chromatograms of standard solutions (25 ng mL^−1^ of each analyte) are gathered in Figure S2, Supplementary Material, showing suitable chromatographic separation in less than 10 min. The partial overlapping of the MAR and LEV signals is not a problem because the two analytes exhibit different fragmentation profiles, which ensure selective and accurate quantification in the MRM mode.

The parameters in the ESI-MS/MS were drying gas (N_2_) temperature 300 °C; drying gas flow 7 L min^−1^; nebulizer 45 psi; sheath gas temperature 350 °C; sheath gas flow 7 L min^−1^; capillary voltage 3500 V positive; nozzle voltage 500 V positive; cell accelerated voltage (CAV) 4 V positive [25].

Quantitative analysis was performed in multiple reaction monitoring (MRM) mode, selecting for each compound [M+H]^+^ as parent ion, the most intense of the transitions being the quantifier ion and the second most abundant of the transitions being qualifier m/z, as reported in Table S1, Supplementary Material [25]

### 3.5. Analytical Evaluation of the Method

The entire analytical method was within-laboratory evaluated, according to the main validation guidelines and criteria for analytical method development in complex matrices [23,26,27,28,29].

In this work, selectivity was evaluated by the analysis of the extracts from freeze-dried food samples with and without analytes’ spike to evaluate the role of co-extracted components from the matrix.

To assess both linearity in the matrix extracts and ME, calibration curves were prepared for each analyte in pure solvent (0.1% HCOOH-ACN 80:20, *v/v*) and in each purified extract from freeze-dried food samples (matrix-matched calibration curves) in the concentration range of 2–25 μg L^−1^, levels corresponding to 25, 50, 75, 100, and 150%, the expected concentrations after UAE-SPE procedure. In ESI, ionization efficiency of the analytes may be strongly altered by the co-eluting compounds, and different matrices can lead to different matrix effects, thus ME was investigated in each purified extract from freeze-dried vegetables and it was calculated as:

(1) $$\left(\%\right)=\left(\frac{bm}{bs}-1\right)\times 100$$

where “*b_m_*” and “*b_s_*” are the slopes of the matrix-matched calibration curve and of the calibration line obtained in pure solvent, respectively [26,27], obtained by ordinary linear lowest squares regression (OLLSR).

MDLs and MQLs (ng g^−1^) were calculated from the matrix-matched calibration curves as 3.3 and 10 times the ratio between the baseline noise away from the peak tail and the regression line slope, respectively, taking into consideration the whole sample treatment [30,31,32].

Independent recovery tests (*n* = 3) were performed on freeze-dried food samples enriched with each analyte at three different concentration levels, namely low-quality control (LQC, 15 ng g^−1^), medium-quality control (MQC, 70 ng g^−1^) and high-quality control (HQC, 150 ng g^−1^), to evaluate trueness and precision. Trueness was calculated as the ratio of the concentration obtained after the entire procedure of the spiked sample and the concentration expected. The inter-day precision was expressed as RSD % associated to the mean recovery.

The stability of FQs in the vegetable samples was determined using samples kept in the dark at room temperature for 30 days and then analyzed.

Robustness was evaluated by small and deliberate variations in operating conditions, whereas instrumental carry-over was checked by injections of SPE eluting solution and SPE eluates from each food extract as control blanks after consecutively analyzed samples.

The repeatability of the injection is one of the parameters included in the System Suitability Test, used to evaluate the accuracy of a chromatographic analysis within an analytical method [28]. It was checked by repeated injections (*n* = 10) of a 15 ng mL^−1^ FQ standard solution prepared both in neat solvent and in the SPE eluate from each freeze-dried food submitted to the definitive analytical procedure.

## 4. Conclusions

The goal of this work is the development of a simple, quick and efficient analytical method for determination of human and veterinary FQ residues in vegetables. The extraction greatly contributes to the greenness of the whole analytical process, as it couples the high-performance and energy-efficient ultrasound instrument with the high stability of the FQ-Mg^2+^ complex, thus not requiring the use of an organic solvent.

Moreover, the combination of the SPE cleanup with HPLC-MS/MS ensures a firm identification and quantitation of the target compounds, with a strong reduction in the volume of organic solvents for cleanup and chromatographic separation, and an accurate determination of FQ traces also at a few nanograms per gram. The method, developed and validated in real matrices, is very attractive compared to the few existing protocols that involve laborious sample preparation and/or the use of large volumes of organic solvents. The applicability to FQ residue monitoring, demonstrated on supermarket lettuce, cucumber and spinach samples, suggests that the procedure may be extended to other green fruits and leafy vegetables, and applied in monitoring campaigns on a wider number of samples.

## Figures and Tables

**Table 1:** Mean recovery values (% R, *n* = 3) with relative standard deviation (RSD%) in freeze-dried samples (spinach, lettuce and cucumber) spiked at three QC levels.

Analyte	Spinach			Lettuce			Cucumber		
	HQC	MQC	LQC	HQC	MQC	LQC	HQC	MQC	LQC
MAR	92(13)	91(6)	92(12)	91(12)	96(2)	92(4)	105(13)	86(13)	106(14)
NOR	80(16)	74(3)	80(12)	81(15)	116(9)	103(5)	100(8)	90(20)	114(4)
LEV	84(9)	84(16)	96(10)	90(3)	106(5)	93(16)	97(8)	88(11)	107(4)
CIP	75(7)	81(7)	91(5)	80(7)	88(18)	90(16)	89(8)	83(19)	111(6)
DAN	75(12)	70(11)	86(9)	95(7)	96(12)	89(12)	88(4)	85(16)	103(3)
ENR	81(10)	73(8)	67(6)	90(2)	105(3)	96(9)	80(15)	86(15)	99(4)
ORB	80(5)	95(8)	81(13)	89(10)	93(18)	94(18)	84(16)	75(5)	100(11)

**Table 2:** MDL and MQL values (ng g^−1^) obtained for each analyte from matrix-matched calibration curves, taking into consideration the whole sample treatment.

Analyte	Spinach		Lettuce		Cucumber	
	MDL	MQL	MDL	MQL	MDL	MQL
MAR	1	4	1	2	2	5
NOR	3	10	2	5	1	3
LEV	2	5	1	2	1	3
CIP	2	5	1	3	2	5
DAN	1	2	1	3	1	2
ENR	1	3	1	2	1	3
ORB	1	3	1	3	1	2

**Table 3:** Comparison with current methods entailing UAE followed by LC-MS quantification of trace FQs in vegetables.

Matrix	Analyte	UAE	Cleanup	Recovery (%)	MQL (ng g^−1^)	Ref.
Cabbage Spinach Radish Corn Rice 1 g (d.w.)	NOR	3 × 15 min × 30 mL acidified ACN-acetone (1:1 *v/v*)	HLB™ SPE on 1:200 diluted extract	81–87	1.17	[18]
Potato Carrot Lettuce Wheat 250 mg (d.w.)	NOR CIP Ofloxacin	1 × 5 min × 10 mL ACN−1% CH_3_COOH (1:1 *v/v*)	HLB™ SPE on 1:15 diluted extract	66–93	5–40	[19]
Lettuce Tomato Cauliflower Bean 1 g (f.w.)	ENR Ofloxacin	2 × 15 min × 10 mL MeOH	Strata™-X SPE on 1:10 diluted extract	30–125	0.4–9.2	[9]
Lettuce 0.2 g (d.w.)	CIP ENR	2 × 15 min × 5 mL ACN-MeOH−0.5% HCOOH (65:15:20, *v/v/v*)	C18 d-SPE	70–90	10–24	[10]
Spinach Lettuce Cucumber 0.25–0.5 (d.w.)	MAR NOR LEV CIP DAN ENR ORB	3 × 2 min × 6 mL 20% *w/v* Mg(NO_3_)_2_ 2% *v/v* NH_3_	HLB™ SPE on ~1:3 diluted extract	67–116	2–10	This work

## Data Availability

The data presented in this study are available on request from the corresponding authors.

## Supplementary Materials

The following supporting information can be downloaded at: https://www.mdpi.com/article/10.3390/molecules27196595/s1, Figure S1: MRM chromatogram (in the time range 6–9.5 min) of purified extract from freeze-dried sample of commercial cucumber (NOR quantified at 130 ng g^−1^, LEV at 4 ng g^−1^ and ENR at 5 ng g^−1^); Figure S2: TIC and MRM chromatograms from an FQ standard mixture in 0.1% HCOOH-ACN, 80:20 *v*/*v* (25 ng mL^−1^ of each analyte); Table S1: MRM conditions for the HPLC-ESI-MS/MS analysis of FQs; Table S2: Calibration curves and correlation coefficients in pure solvent; Table S3: Calibration curves and correlation coefficients in purified extract from freeze-dried spinach; Table S4: Calibration curves and correlation coefficients in purified extract from freeze-dried lettuce; Table S5: Calibration curves and correlation coefficients in purified extract from freeze-dried cucumber.

## Sample Availability

Samples of the compounds are not available from the authors.
