# Supplementary material for: Green and Efficient Determination of Fluoroquinolone Residues in Edible Green Fruits and Leafy Vegetables by Ultrasound-Assisted Extraction Followed by HPLC-MS/MS
Source: Molecules. 2022 Oct 5;27(19):6595. doi: 10.3390/molecules27196595 (PMC9572745; doi:10.3390/molecules27196595)
Supplement: Supplementary file 1 [file molecules-27-06595-s001.zip › molecules-1940355-supplementary.pdf]

Supplementary Material

Green and Efficient Determination of Fluoroquinolone Residues in Edible Green Fruits and Leafy Vegetables by Ultrasound-Assisted Extraction Followed by HPLC-MS/MS

Francesca Merlo [1,*], Dario Centenaro [2], Federica Maraschi [1], Antonella Profumo [1] and Andrea Speltini [1,*]

[1] Department of Chemistry, University of Pavia, 27100 Pavia, Italy
[2] Department of Drug Sciences, University of Pavia, 27100 Pavia, Italy
* Correspondence: francesca.merlo@unipv.it (F.M.); andrea.speltini@unipv.it (A.S.); Tel.: +39-0382-98-73-50 (A.S.)

Table S1. MRM conditions for the HPLC-ESI-MS/MS analysis of FQs.

Analyte	Precursor Ion (m/z)	Product Ion (m/z)	Fragmentor (V)	Collision Energy (V)	Dwell Time (ms)
MAR	363.0	345.1	130	21	75
		320.0	130	13	75
LEV	362.0	318.1	140	21	75
		261.0	140	29	75
NOR	320.0	302.1	130	25	75
		276.1	130	17	75
CIP	332.1	314.1	130	21	75
		231.1	130	40	75
DAN	358.1	340.1	135	25	75
		82.1	135	49	75
ENR	360.1	342.1	130	25	75
		316.1	130	21	75
ORB	396.2	352.2	102	25	75
	396.2	295.0	102	33	75
ENOX	321.1	303.1	125	16	75
	321.1	232.1	125	36	75

Table S2. Calibration curves and correlation coefficients in pure solvent.

Analyte	Calibration line equation*	R^2
MAR	y = 1451 (57) x + 1304 (724)	0.9959
NOR	y = 2089 (67) x + 1870 (860)	0.9964
LEV	y = 4329 (130) x + 3570 (1660)	0.9959
CIP	y = 2103 (87) x + 2652 (1110)	0.9932
DAN	y = 2404 (144) x + 4280 (1835)	0.9920
ENR	y = 2367 (69) x + 1804 (881)	0.9966
ORB	y = 4633 (166) x + 1597 (2126)	0.9949

* Calculated as peak areas vs. concentration; in parentheses the uncertainties associated to slope and intercept of the mean calibration curve obtained from Ordinary Linear Lowest Square Regression (OLLSR).

Table S3. Calibration curves and correlation coefficients in purified extract from freeze-dried spinach.

Analyte	Calibration line equation*	R^2
MAR	y = 431 (7) x + 80 (92)	0.9989
NOR	y = 529 (3) x + 349 (40)	0.9993
LEV	y = 1262 (16) x + 192 (207)	0.9999
CIP	y = 800 (8) x + 57 (96)	0.9996
DAN	y = 3166 (35) x + 624 (442)	0.9995
ENR	y = 1392 (21) x + 181 (264)	0.9991
ORB	y = 558 (6) x + 197 (81)	0.9995

* Calculated as peak areas vs. concentration; in parentheses the uncertainties associated to slope and intercept of the mean calibration curve obtained from Ordinary Linear Lowest Square Regression (OLLSR).

Table S4. Calibration curves and correlation coefficients in purified extract from freeze-dried lettuce.

Analyte	Calibration line equation*	R^2
MAR	y = 733 (11) x - 38 (92)	0.9992
NOR	y = 1003 (21) x + 112 (264)	0.9967
LEV	y = 2204 (63) x - 175 (810)	0.9983
CIP	y = 3371 (111) x - 417 (1420)	0.9957
DAN	y = 7162 (168) x - 529 (2142)	0.9978
ENR	y = 2145 (54) x - 376 (683)	0.9975
ORB	y = 1327 (47) x - 239 (607)	0.9949

* Calculated as peak areas vs. concentration; in parentheses the uncertainties associated to slope and intercept of the mean calibration curve obtained from Ordinary Linear Lowest Square Regression (OLLSR).

Table S5. Calibration curves and correlation coefficients in purified extract from freeze-dried cucumber.

Analyte	Calibration line equation*	R^2
MAR	y = 777 (15) x + 201 (195)	0.9985
NOR	y = 1155 (40) x + 135 (510)	0.9952
LEV	y = 2481 (86) x + 45 (1101)	0.9953
CIP	y = 4630 (203) x + 483 (2597)	0.9924
DAN	y = 9702 (421) x + 1322 (5381)	0.9925
ENR	y = 2592 (79)x + 47 (1009)	0.9963
ORB	y = 1762 (67) x - 310 (850)	0.9943

* Calculated as peak areas vs. concentration; in parentheses the uncertainties associated to slope and intercept of the mean calibration curve obtained from Ordinary Linear Lowest Square Regression (OLLSR).

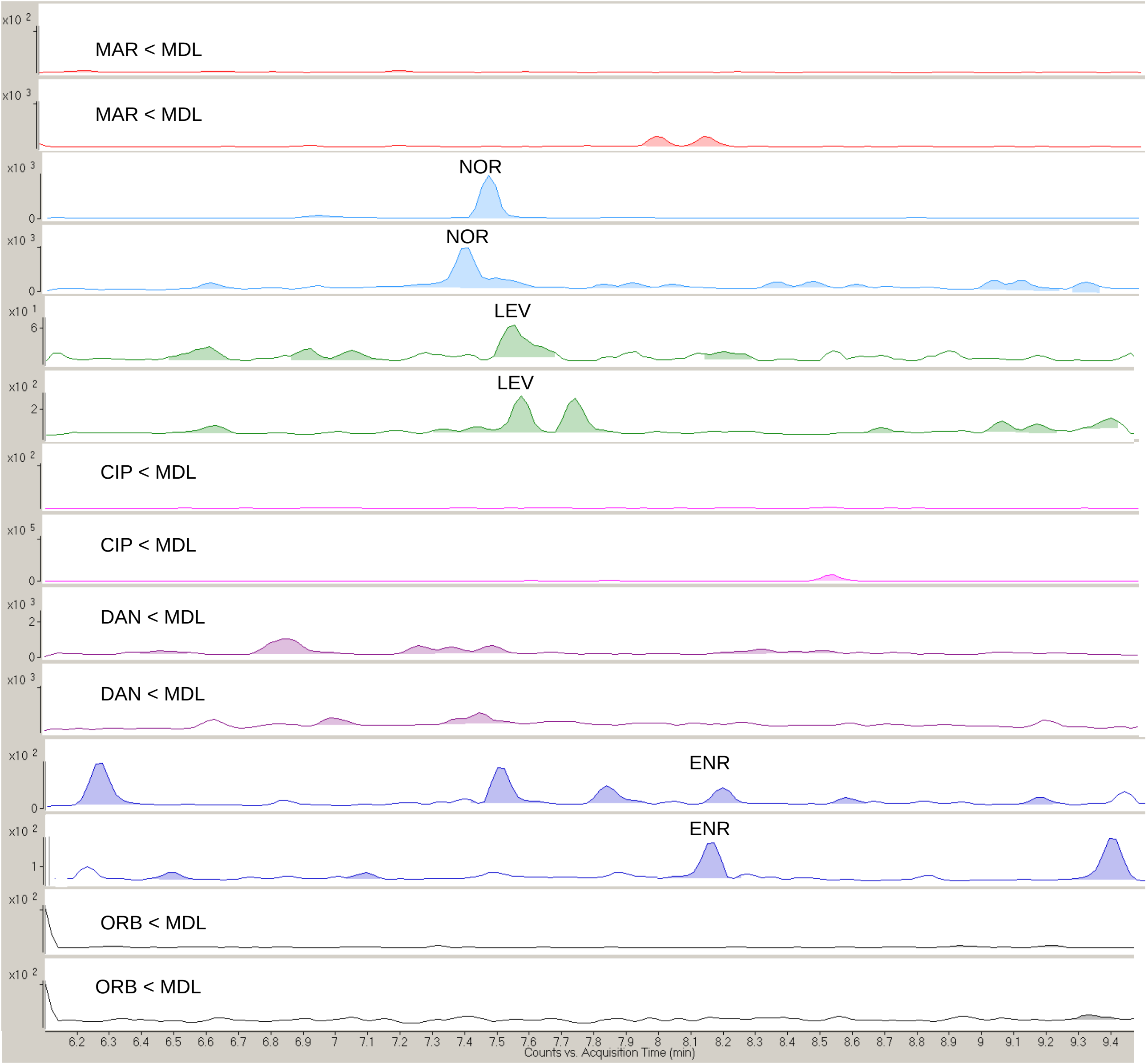

Figure S1. MRM chromatogram (in the time range 6–9.5 min) of purified extract from freeze-dried sample of commercial cucumber (NOR quantified at 130 ng g^{-1}, LEV at 4 ng g^{-1} and ENR at 5 ng g^{-1}).

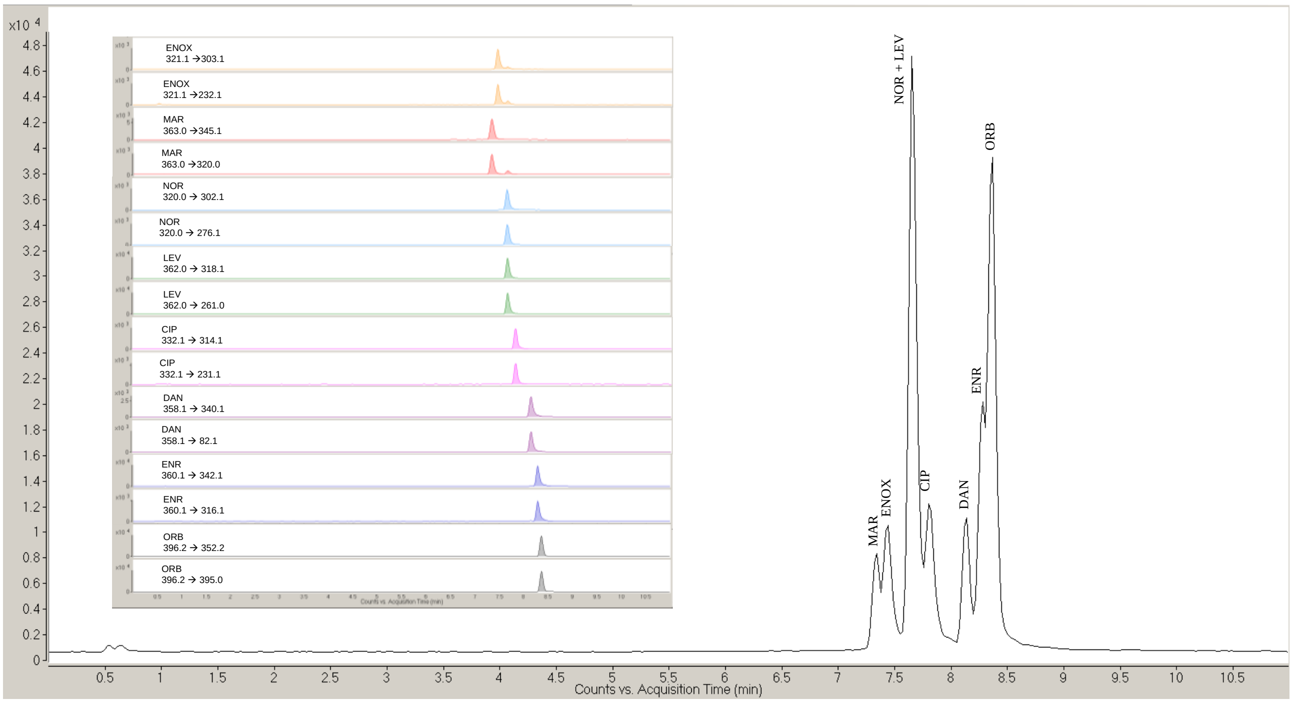

Figure S2. TIC and MRM chromatograms from a FQs standard mixture in 0.1% HCOOH-ACN, 80:20 *v*/*v* (25 ng mL^{-1} of each analyte).
